# Animal Models and Molecular Pathogenesis of Arrhythmogenic Cardiomyopathy Associated with Pathogenic Variants in Intercalated Disc Genes

**DOI:** 10.3390/ijms25116208

**Published:** 2024-06-05

**Authors:** Sara Vencato, Chiara Romanato, Alessandra Rampazzo, Martina Calore

**Affiliations:** 1 Department of Biology, University of Padova, Via U. Bassi 58/B, 35121 Padova, Italy; sara.vencato.1@phd.unipd.it (S.V.); chiara.romanato@phd.unipd.it (C.R.); alessandra.rampazzo@unipd.it (A.R.); 2 Department of Molecular Genetics, Faculty of Health, Medicine and Life Sciences, Maastricht University, 6211 LK Maastricht, The Netherlands

**Keywords:** arrhythmogenic cardiomyopathy, intercalated disc, murine models, pathogenic mechanisms

## Abstract

Arrhythmogenic cardiomyopathy (ACM) is a rare genetic cardiac disease characterized by the progressive substitution of myocardium with fibro-fatty tissue. Clinically, ACM shows wide variability among patients; symptoms can include syncope and ventricular tachycardia but also sudden death, with the latter often being its sole manifestation. Approximately half of ACM patients have been found with variations in one or more genes encoding cardiac intercalated discs proteins; the most involved genes are plakophilin 2 (*PKP2*), desmoglein 2 (*DSG2*), and desmoplakin (*DSP*). Cardiac intercalated discs provide mechanical and electro-metabolic coupling among cardiomyocytes. Mechanical communication is guaranteed by the interaction of proteins of desmosomes and adheren junctions in the so-called *area composita*, whereas electro-metabolic coupling between adjacent cardiac cells depends on gap junctions. Although ACM has been first described almost thirty years ago, the pathogenic mechanism(s) leading to its development are still only partially known. Several studies with different animal models point to the involvement of the Wnt/β-catenin signaling in combination with the Hippo pathway. Here, we present an overview about the existing murine models of ACM harboring variants in intercalated disc components with a particular focus on the underlying pathogenic mechanisms. Prospectively, mechanistic insights into the disease pathogenesis will lead to the development of effective targeted therapies for ACM.

## 1. Introduction

Arrhythmogenic cardiomyopathy (ACM, OMIM #107970) is a rare cardiac genetic disease recognized as one of the leading causes of sudden cardiac death (SCD) in young people and athletes [1]. The pathological hallmark of ACM is the progressive loss of the myocardium and its replacement by fibrofatty tissue, starting from the epicardium or midmyocardium and then extending to become transmural [2]. The diffused or regional infiltration is located at the angles of the so-called “triangle of dysplasia”, including the inferior, apical, and infundibular walls of the heart [2].

ACM is mostly inherited as an autosomal dominant condition, and its prevalence is estimated to be 1:2000–1:5000 [1]. The disease presents variable expressivity and reduced age-related penetrance [3]. Approximately half of ACM patients present pathogenic variants in one or more genes encoding cardiac intercalated disc (ID) proteins, which provide mechanical and electro-metabolic coupling among cardiomyocytes [4]. The most commonly affected genes are those coding for desmoplakin (*DSP*), plakophilin 2 (*PKP2*), desmoglein 2 (*DSG2*), desmocollin 2 (*DSC2*), and plakoglobin (*JUP*) [5]. Additional ACM pathogenic variations have been found in genes encoding αT-catenin (*CTNNA3*), N-cadherin (*CDH2*), transmembrane protein-43 (*TMEM43*), and zonula occludens-1 (*TJP1*) [6,7]. These proteins are mechanical components of intercalated discs, involved in maintaining the mechanical interaction among neighboring cells. Less frequently, ACM pathogenic variations have been detected in genes encoding non-junctional proteins, such as the ryanodine receptor 2 (*RYR2*), transforming growth factor β3 (*TGFB3*), desmin (*DES*), titin (*TTN*), lamin A/C (*LMNA*), phospholamban (*PLN*), and sodium voltage-gated channel alpha subunit 5 (*SCN5A*) [7]. Although most of these non-junctional proteins are not part of intercalated discs, they are located in close proximity, and they often establish a direct or indirect interaction with ID proteins.

Besides the above-mentioned genetic determinants, many other environmental factors largely impact ACM onset, progression, and prognosis. In particular, exercise is known to trigger life-threatening ventricular arrhythmias [8]; accordingly, the risk of sudden cardiac death is increased up to five-fold in ACM-affected athletes and arrhythmic events often occur during exercise [9,10].

Although ACM was first described more than thirty years ago, a detailed comprehension of the pathogenic mechanisms leading to its development is still missing. Several studies based on different animal models point to the inhibition of the Wnt/β-catenin signaling in combination with the activation of the Hippo and TGFβ pathways as key players in ACM physiopathology by regulating the adipogenic and fibrotic cascades [3]. In this review, we present an overview about the existing murine models of ACM harboring pathogenic variants in intercalated disc components with a particular focus on the underlying pathogenic mechanisms.

## 2. Intercalated Discs

**Figure 1:**
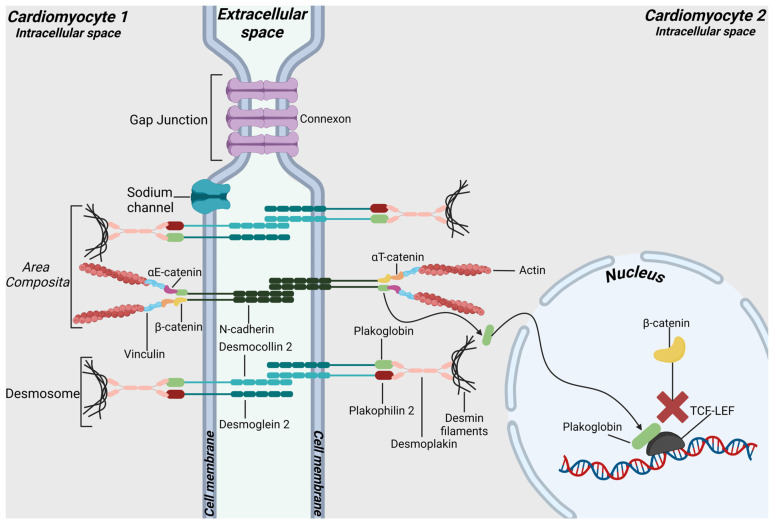
Molecular organization of junctions at intercalated discs (IDs). Major components of desmosomes are desmoglein 2 (DSG2) and desmocollin 2 (DSC2), which connect neighboring cells through their extracellular domains. Intracellularly, these cadherins are linked to the armadillo proteins plakophilin 2 (PKP2) and plakoglobin (PG), which are in turn connected to desmoplakin (DSP). Finally, DSP anchors the desmosomal plaque to desmin intermediate filaments (IF). The *area composita* is a heart-specific hybrid junction composed by proteins of desmosomes and adherens junctions (AJ), such as β-catenin, N-cadherin, and αT-catenin. The latter binds the F-actin cytoskeleton directly or indirectly, via vinculin. Gap junctions, mainly consisting of connexin 43 (CX43), and ion channels represent the electrical components of the ID and are crucial for the electrical coupling of the heart. In the pathogenesis of ACM, mutations in genes encoding junctional proteins can induce an important “domino effect” with the consequent loss of cardiomyocytes adhesion. These structural changes prevent the integration in the ID of PG, which translocates into the nucleus and inhibits the canonical Wnt/β-catenin signaling by binding TCF/LEF transcriptional factors.

The mechanical, chemical, and electrical coupling between adjacent myocardial cells is ensured by IDs, highly specialized structures present in the heart between the cardiomyocytes [4]. These intercellular junctions include desmosomes and *area composita*, with the latter comprising elements of desmosomes and adherens junctions (AJ), as well as gap junctions and ion channels, which facilitate electrical communication [4] (Figure 1).

Cardiac desmosomes are multi-protein complexes composed of cadherins, armadillo proteins, and plakins. In this context, the transmembrane cadherins desmocollin 2 (DSC2) and desmoglein 2 (DSG2) form extracellular Ca^2+^-dependent homophilic and heterophilic connections with cadherins protruding from the surface of neighboring cells [11]. Both cadherins are characterized by five extracellular domains (EC1-5), a single transmembrane portion, and an intracellular C-terminal tail [11]. At the cytosolic level, the armadillo proteins plakoglobin (PG) and plakophilin 2 (PKP2) connect transmembrane cadherins to desmoplakin (DSP), which in turn binds the intermediate filaments (IF) of desmin of the cytoskeleton [12].

AJs are also mechanical multi-protein complexes, and they include the transmembrane protein N-cadherin, whose extracellular domains mediate cell–cell adhesion with another cadherin molecule exposed by a neighboring cell [11]. On the intracellular side, AJs are anchored to actin filaments of cytoskeleton thanks to the interaction of N-cadherin with p120 and β-catenin, both members of the armadillo repeat protein family, and other structural proteins such as vinculin, αE-catenin, and αT-catenin. In this way, AJs provide continuity to actin filaments from adjacent cardiomyocytes, thus contributing to the transmission of the contractile force between cardiomyocytes [9]. Interestingly, in the postnatal mammalian heart, desmosomes and AJs do not exist as distinct entities, but they are almost completely present in *area composita* [13]. In this hybrid junction, typical desmosomal proteins are found in proximity to proteins of AJs, such as β-catenin, N-cadherin, and αT-catenin, which, by binding PKP2, provide a direct link between the cadherin/catenin complex and the actin cytoskeleton [5]. Being interrupted only by few genuine desmosomes, gap junctions, and junction-free segments, *area composita* represents the largest portion of ID (>90%) [13].

Desmosomes and *area composita* fulfil the role of mechanical junctions and are particularly important for the maintenance of the integrity and cell adhesion of the myocardial tissue, constantly exposed to mechanical stress [5].

Gap junctions (GJ) allow the passage of small molecules and the propagation of action potential between cardiomyocytes [14]. GJ plaques are clusters of multiple channels, known as connexons, which are composed of six integral membrane proteins referred to as connexins. The prevalent connexin expressed in the mammalian heart is connexin 43 (CX43) [11].

Along with GJ, ion channels, spanning the membrane of cardiac cells, are important for the generation and transmission of contractile impulse throughout the heart [11]. Voltage-dependent potassium and sodium channels are involved in repolarization and depolarization, respectively. Alterations in their functionality can cause defects in electrical propagation among cardiomyocytes, possibly leading to cardiac diseases [11].

Besides their structural role, ID proteins also participate in signaling pathways [15]. For example, both mechanical junction proteins β-catenin and PG are key players of the canonical Wnt/β-catenin signaling pathway, which regulates embryonic heart development and adult tissue homeostasis [16] (Figure 1). Activation of this pathway allows unphosphorylated β-catenin to translocate into the nucleus, where it interacts with TCF/LEF transcription factors, leading to the expression of genes promoting cell proliferation and differentiation [17]. Conversely, in the absence of Wnt ligands, the destruction complex, including GSK3β, APC, AXIN, and CK1, promotes β-catenin phosphorylation and subsequent ubiquitination and degradation via the proteasome [16]. Studies on various ACM models suggest that pathogenic variants in desmosomal genes may cause not only structural alterations in ID but also the migration of PG into the nucleus. Here, PG competes with its homologous β-catenin for binding to TCF/LEF, eventually suppressing the canonical Wnt signaling pathway [18].

## 3. Murine Models for ACM Associated with Plakophilin 2 Variants

*PKP2* is identified as the most commonly mutated gene in ACM patients, with pathogenic variants in *PKP2* being detectable in 20–46% of affected individuals [7]. The observed reduction of PKP2 in cardiac biopsies from ACM patients suggests that variations in this gene commonly lead to haploinsufficiency, potentially resulting in ACM [19,20].

Encoded by the *PKP2* gene, PKP2 is a structural ID protein belonging to the armadillo protein family [11]. It comprises eight arm repeats involved in the anchorage of IF by interacting with cadherins and desmoplakin [21].

The crucial role of PKP2 was demonstrated by loss of function studies in mice, which died in the embryonic stage when carrying the null allele in homozygosity [22] (Table 1). At E10.5, these embryos showed mislocation of typical desmosomal proteins such as DSG2, DSP, and PG, along with altered organization of junctions between cardiomyocytes [22]. The conclusion of the authors on the central role of PKP2 in cytoskeletal and junctional stability in the myocardium was further validated by ultrastructural analysis performed in a *Pkp2* heterozygous null murine model presenting reduction in the number of mechanical junctions together with a decrease in the intercellular distance in correspondence of the IDs [23] (Table 1). Furthermore, molecular investigations were performed on cardiac samples from the same heterozygous mice to study the underlying pathogenic mechanisms. Western blot analyses revealed that loss of PKP2 activated the profibrotic TGF-β1/p38 MAPK signaling pathway, resulting in the upregulation of profibrotic cytokines and extracellular matrix (ECM) components [23,24] (Table 1). Studies conducted by Cerrone and colleagues on the same heterozygous *Pkp2* knockout mice reported the dysregulation of the Na_v_1.5 sodium channel upon PKP2 haploinsufficiency [25] (Table 1). Interestingly, the Na_v_1.5 channel was shown to interact with CX43 and PKP2, highlighting the crosstalk between proteins of mechanical and electrical junctions in the heart [25]. To investigate deeper into the role of PKP2 in ACM pathogenesis, the same group generated a cardiomyocyte-specific *Pkp2* conditional knockout murine model (PKP2cKO) exhibiting cardiac fibrosis, ventricular dysfunction, and arrhythmias. Transcriptomic analysis suggested that loss of *Pkp2* may alter intracellular calcium homeostasis, predisposing to arrhythmias [26] (Table 1). Subsequent analyses on the same model were conducted to validate this hypothesis and evaluate the role of reduced *Pkp2* expression in the arrhythmogenic aspect of the disease [27]. In vitro studies on cardiomyocytes isolated from the right and left ventricular free wall of the heart of PKP2cKO mice revealed enhanced frequency of spontaneous calcium release events, along with increased calcium concentration in the cytoplasm of cells from the right ventricle [27]. Based on the important role of GJs in the electrochemical coupling between neighboring cardiomyocytes function, the authors hypothesized that alterations in the expression and activity of CX43 can be associated with the observed alterations in calcium levels [27]. To test this hypothesis, Kim and colleagues performed additional experiments on cardiomyocytes isolated from the cardiac tissue of PKP2cKO mice crossed with heterozygous conditional knockout for cardiac CX43. Intracellular calcium levels were comparable to those measured in cardiomyocytes extracted from the heart of WT animals. These results led the authors to speculate that loss of PKP2 might cause an excess of “orphan” CX43 hemichannels, allowing calcium entry into cardiomyocytes, even not integrated into GJ plaques [27] (Table 1). Modifications in the localization of CX43, coupled with right ventricle dysfunction, were also noted in a transgenic mouse model overexpressing the human *PKP2* gene variant c.2203C>T (p.R375*) [28] specifically in cardiomyocytes [29] (Table 1). Similar to observations in humans, these phenotypic alterations were not observed in resting animals but were induced by endurance exercise. Also, the p.S329* murine variant was studied in transgenic models [30] (Table 1). In this case, three transgenic lines expressing increasing levels of this truncated form of PKP2 were created: TG-L mice with low mutant *Pkp2* expression; TG-M models, presenting a medium expression of the truncated protein; and TG-H animals, with high expression of the transgene. The severity of ACM features was transgene level dependent [30]. Mutant animals presenting the highest expression of the transgene exhibited, compared to WT controls, ventricular arrhythmias, as well as important reduction in left ventricular systolic function together with alterations of the right ventricle function. Conduction alterations were related to reduced expression of proteins associated to electrical coupling, such as CX43 and the sodium channel subunit Na_v_1.5, which was observed also in TG-M animals. Furthermore, high levels of truncated PKP2 resulted in loss of cardiomyocyte adhesion; downregulation of PG, DES, and β-catenin; and disruption of ID structures [30]. Ultrastructural analyses did not detect any ID alteration in TG-M mice compared to WT animals. On the contrary, alterations in the function of the right ventricle were evident in both TG-M and TG-L lines, even if in a lower extent compared to TG-H mice [30]. Altogether, these results indicate that overexpression of mutant *Pkp2* might have a dose-dependent pathogenic effect in mice and that this protein has a possible role in predisposition to arrhythmia.

Camors and colleagues generated a heterozygous knock-in murine model for the c.1086InsT frameshift variant, corresponding to the human c.1211dupT variant [31], which showed a prevalent involvement of the right ventricle. On the other hand, the absence of the mutated gene and protein probably explains the early lethality of homozygous embryos [32] (Table 1). The heterozygous animals during their first year of life showed a progressive decline of the right ventricle function, correlated with reduction in ventricular cardiomyocyte function, as demonstrated with in vitro tests [32]. Nonetheless, molecular analyses did not reveal any alteration in the expression of sarcomere genes and proteins, suggesting that reduced PKP2 level itself affects sarcomere function [32].

In the majority of the models described above, it was highlighted that haploinsufficiency of *Pkp2* leads to the manifestation of phenotypical and functional features typical of ACM. This concept was further supported by a recent heterozygous knock-in mouse model harboring the *Pkp2* c.1755delA alteration, corresponding to the human variant c.2013delC [33] (Table 1). Mutant animals showed cardiac fibrosis and disorganization of junctional structures characterized by increased width of IDs together with important reduction in the expression of junctional proteins such as PKP2, JUP, DSP, DSG, β-catenin, and N-cadherin at 12 months of age [33]. Proteomics analyses on these animals detected the enhanced expression of proteins associated to the ubiquitin–proteasome system (UPS), suggesting the involvement of this system in the degradation of *area composita* and desmosomal proteins. Accordingly, treatment of affected animals with MG132, an inhibitor of proteasome, rescued the expression of PKP2, JUP, and DSP, opening new possibilities for therapeutic approaches [33].

Another recent study suggested that splicing aberrations can also result in ACM [34] (Table 1). The knock-in homozygous murine model carrying the splicing variant equivalent to the human c.2014-1G>C variation [35] manifested electrical dysfunction and sudden death starting at 4 weeks of age [34]. Electrophysiological and immunohistochemical analyses on these mice revealed both alterations in the sodium current and loss of the Na_v_1.5 subunit at the ID, corroborating the crosstalk between PKP2 and electrical components. Additionally, transmission electron microscopy performed on the hearts of the same animals at 2 weeks of age revealed reduction in the number of desmosomes, when compared to control mice [34]. Moreover, histological analysis performed on 6-week-old animals detected enlarged ventricles, loss of myocardium, and fibrous replacement. Interestingly, lipid accumulation in the subepicardium of the right ventricle of these animals was also detected. The homozygous mutation resulted also in decreased levels of DSP, DSG2, JUP, and CX43, and surprisingly a mutant PKP2 with a higher molecular weight was detected, suggesting that the mutation in the splice acceptor site determines the use of an alternative splice site with the intron retention [34].

The studies described above have yet to fully clarify the regulatory mechanisms of pathways involved in the pathogenesis of ACM. Approaching the issue from a fresh angle, Gurha and colleagues proposed that microRNAs may target the molecular signals underlining ACM [36] (Table 1). Specifically, the authors focused on the role of miR-184, which was found downregulated in the heart of a murine model in which the expression of *Pkp2* was knocked down. These animals showed enlarged left ventricle, interstitial fibrosis, and the presence of adipocytes [36]. Complementary in vitro studies on PKP2-deficient HL-1 cardiomyocytes revealed increased expression of adipogenic markers along with fat droplet accumulation upon suppression of miR-184. In contrast, the rescue of the adipogenic phenotype was obtained in these cells by the overexpression of the same microRNA. Nonetheless, alteration of microRNA expression did not have any effect on the Hippo and Wnt/β-catenin signaling pathways [36].

Altogether, these studies highlight the crucial role of PKP2 in the pathogenesis of ACM and in maintaining gap junction integrity, leading to the hypothesis that loss of PKP2 predisposes to arrhythmias in patients.

**Table 1:** PKP2 murine models for ACM and corresponding features.

Gene	Experimental Model	Alterations	Refs.
*Pkp2*	*Pkp2* homozygous-null embryos	Mislocation of desmosomal proteins Altered organization of cardiac junctions	[22]
	*Pkp2* heterozygous-null mice	Reduced number of mechanical junctions Reduced intercellular distances in correspondence of IDs Upregulation of ECM components ↑TGF-β1/p38 MAPK pathway Desmosomes sporadic or absent Expanded space between cardiomyocytes Ventricular arrhythmias	[23,24,25]
	Cardiomyocyte-specific *Pkp2* conditional KO	Right ventricle dysfunction Arrhythmias Cardiac fibrosis Increased calcium concentration Enhanced frequency of spontaneous calcium release events measured in vitro	[26,27]
	Complete *Pkp2* knock-down via shRNA	Enlarged left ventricle Interstitial fibrosis Presence of cells with lipid droplets	[36]
	Tg mice with cardiomyocyte-specific overexpression of PKP2 with p.R375* variation	Right ventricle dysfunction exacerbated by training Altered localization and distribution of CX43	[29]
	Tg mice with cardiomyocyte-specific overexpression of PKP2 with p.S329* variation	Ventricular arrhythmias Reduced left ventricle systolic function Reduced expression of proteins CX43 and Na_V_1.5 Downregulation of junctional proteins expression	[30]
	*Pkp2* heterozygous KI mice with c.1086InsT variation	Reduced right ventricle functionality Increased apoptotic cardiomyocytes Increased widening of intercalated discs	[32]
	*Pkp2* heterozygous KI mice with c.1755delA variation	Cardiac fibrosis Reduced expression of junctional proteins Increased width of IDs	[33]
	*Pkp2* homozygous KI mice	Alterations in sodium current Loss of Na_v_1.5 subunit Enlarged ventricles Cardiac fibrosis Subepicardial adiposis Reduced number of desmosomes Reduced expression of junctional proteins	[34]

## 4. Murine Models for ACM Associated with Desmoplakin Variants

Pathogenic *DSP* variations can be identified in 3–20% of all ACM patients [7]. Additionally, autosomal recessive inheritance of variations in *DSP* is also causative of Carvajal syndrome, characterized by keratoderma, woolly hair, and cardiac manifestations predominantly involving the left ventricle [6].

DSP is a member of the plakin family of cytoskeleton-associated proteins. It consists of a structural protein that forms dimers and links desmosomes to desmin IF, thus being critical for normal force transmission in the myocardium [37].

The crucial role of DSP in desmosomes assembly, stability, and connection to IF has been underscored by the fact that systemic *Dsp*-deficient mice could not be generated due to early embryonic lethality [38] (Table 2). Similarly, homozygous deletion of the second exon of *Dsp* restricted to cardiomyocytes (*Dsp*^−/−^) caused a high rate of embryonic lethality, with embryos exhibiting poorly formed hearts [39]. *Dsp*^−/−^ mice that survived the embryonic period died within the first two weeks of life and showed extensive fibrotic deposition [39]. Interestingly, cardiomyocyte-specific heterozygous *Dsp* knockout mice (*Dsp*^+/−^) exhibited myocardial dysfunction and arrhythmias linked to excessive fibro-fatty replacement of the myocardium, increased cardiomyocyte death, and poorly organized myocytes [39] (Table 2). Development of ACM features in *Dsp*^+/−^ mice resulted concomitant to the delocalization of PG to the nucleus where it competed with β-catenin. In turn, this could suppress the canonical Wnt signaling pathway promoting the adipogenic switch. Accordingly, reduced expression of downstream Wnt target genes, increased expression of transcriptional regulators of adipogenesis, and accumulation of fat droplets were observed in both *Dsp*^+/−^ mice and *Dsp*-deficient HL-1 cells [39].

Moreover, additional studies on *Dsp*^+/−^ mice have identified the activation of the Hippo pathway as a possible mechanism for the pathogenic suppression of the canonical Wnt signaling in ACM [40]. The Hippo pathway regulates cell proliferation and differentiation through the transcriptional coactivators YAP/TAZ, which translocate into the nucleus and activate their target genes by binding the transcriptional factor TEAD [41]. When the pathway is active, the kinase LATS1/2, activated by phosphorylated neurofibromin 2 protein (NF2), phosphorylates YAP and TAZ, preventing them from activating TEAD [41]. Conversely, phosphorylated YAP binds and sequesters β-catenin at the cell membrane, suppressing the canonical Wnt/β-catenin signaling [40]. Accordingly, in *Dsp*^+/−^ mice, impaired assembly of IDs activated NF2, leading to phosphorylation of YAP and membrane localization of both pYAP and β-catenin, finally resulting in the reduction of both β-catenin/TCF and YAP/TEAD transcriptional activities and enhanced heart adipogenesis [40] (Table 2).

Further studies investigated the transcriptome of cardiomyocytes isolated from the same heterozygous murine model or WT mice and demonstrated the activation of processes related to inflammation and epithelial to mesenchymal transition (EMT) as well as the suppression of the oxidative phosphorylation in cardiac myocytes from *Dsp*^+/−^ mice [42]. Surprisingly, treadmill exercise restored transcript levels of most dysregulated genes, reduced apoptosis, and stimulated cardiac hypertrophy. However, no beneficial effects on cardiac dilatation, dysfunction, or arrhythmias were induced [42] (Table 2).

Lyon and colleagues generated a similar cardiomyocyte-specific *Dsp* homozygous knockout murine model (*Dsp*-cKO) [43]. In this case, mice were viable but showed ultrastructural defects in desmosomal integrity, cardiac cell death, and fibro-fatty replacement leading to biventricular dysfunction, heart failure, and premature death. Also, ventricular arrhythmias were exacerbated with exercise and catecholamine stimulation, supporting the idea that physical effort can aggravate ACM development in rodents [43]. Of note, conduction abnormalities resulting from connexin 40 (CX40) and CX43 downregulation emerged prior to damage in mechanical junctions and fibro-fatty replacement [43]. Neither junctional loss and differences in levels of PG nor Wnt/β-catenin signaling involvement were reported in this model [43] (Table 2). On the other hand, perturbation of the canonical Wnt signaling, increased fibro-adipogenesis in the heart, and mild cardiac dysfunction upon DSP loss were observed also in mice with a conditional heterozygous deletion of the second exon of *Dsp* restricted to cardiac fibro-adipogenic progenitor cells (cFAPs) [44]. The same study allowed also for the demostration that cFAPs express desmosomal proteins and differentiate to adipocytes through a Wnt-dependent mechanism [44] (Table 2).

Yang and colleagues developed different transgenic mice with cardiac restricted overexpression of human WT or mutant forms of DSP [45]. Overexpression of either the N-terminal p.V30M or p.Q90R DSP variants (corresponding to c.88G>A and c.269A>G genetic alterations, respectively [46,47]) resulted in embryonic lethality likely due to abnormalities during heart development [45]. On the other hand, overexpression of the human C-terminal DSP p.R2834H variant, corresponding to the c.8501G>A genetic alteration [48], led to the successful generation of viable transgenic mice, known as R3834H-Tg. These animals exhibited marked alteration of the IDs, cardiac fibrosis, lipid accumulation, and both ventricular enlargement and biventricular cardiomyopathy [45] (Table 2). Moreover, when subjected to endurance exercise, these mice displayed accelerated pathogenesis, in combination with the formation of cytoplasmatic aggregates including DSP, PG, and CX43. The sequestration of PG within these aggregates likely contributed to the reduction of Wnt and Akt1 signaling in exercised R3834H-Tg mice, thereby supporting the shift to adipogenesis [49] (Table 2).

More recently, a new knock-in murine model, named *Dsp*^R451G^, was developed by the introduction in the endogenous *Dsp* gene of the sequence encoding p.R464G variation, the murine equivalent of the human p.R451G (c.1351C>G [50]) variant [51]. While homozygous mice (*Dsp*^R451G/R451G^) displayed embryonic lethality, heterozygous *Dsp*^R451G/+^ mice were viable and showed no structural or functional alterations under baseline conditions. However, these animals showed reduced cardiac performance and increased chamber dilation in response to pressure overload with concomitant prolonged arrhythmic events occurring following catecholaminergic stimulation [51]. Moreover, *Dsp*^R451G/+^ mice exhibited normal expression and distribution of PKP2 and β-catenin; however, minor alterations in CX43 localization were observed at baseline, which became more pronounced following pressure overload [51] (Table 2).

**Table 2:** DSP murine models for ACM and corresponding features.

Gene	Experimental Model	Alterations	Refs.
*Dsp*	General *Dsp* KO mice	Embryonic lethality	[38]
	Cardiomyocyte-specific *Dsp* deficient mice	High embryonic lethality in *Dsp*^−/−^ mice Molecular remodeling of IDs Fibro-fatty replacement LV dilation Reduced fractional shortening Nuclear localization of plakoglobin ↑ Adipogenesis and fibrogenesis ↓ Wnt/β-catenin signaling ↑ Hippo pathway ↑ Inflammation and EMT-related genes ↓ Oxidative phosphorylation-related genes	[39,40,42]
	Cardiomyocyte-specific *Dsp* KO mice	Structural defects in desmosomal integrity Cardiac cells death Fibro-fatty replacement Ventricular arrhythmias exacerbated with exercise or catecholamine stimulation ↓ CX40 protein level ↓CX43 protein level	[43]
	Mice with conditional heterozygous *Dsp* deletion in cFAPs	Increased fibro-adipogenesis Mild cardiac dysfunction ↓ Wnt/β-catenin signaling ↑ Adipogenesis in cFAPs	[44]
	Tg mice with cardiomyocyte-specific overexpression of DSP with p.R3834H variation	Increased cardiomyocytes apoptosis Cardiac fibrosis and lipid accumulations Ultrastructural alterations of IDs Cardiac hypertrophy ↑ PKP2 and β-catenin Accelerated ACM pathogenesis when exposed to endurance exercise ↓ Wnt/β-catenin signaling when exposed to endurance exercise	[45,49]
	*Dsp* KI mice	Embryonic lethality in *Dsp* ^R451G/R451G^ Aberrant CX43 localization Stress-induced arrhythmias Accelerated heart failure following pressure overload	[51]

## 5. Murine Models for ACM Associated with Desmoglein 2 Variants

Genetic variations in the desmoglein 2 gene (*DSG2*) account for 3 to 20% of ACM cases [7]. Most of these variants concentrate in the extracellular portion of the protein and are associated to high pathogenicity, possibly related to the important role of this region for DSG2 interaction between adjacent cardiomyocytes [52].

DSG2 is a transmembrane cadherin found in both desmosomes and *area composita* [9]. Thanks to its extracellular domains, DSG2 forms either Ca^2+^-dependent homophilic connections or heterophilic interactions with the other cadherin DSC2, while in the cytosol, the C-terminal tail interacts with the armadillo proteins PG and PKP2 [9].

In mice, deletion of exons 7 and 8 from the *Dsg2* gene led to the production of a dysfunctional protein terminating at the level of the extracellular EC2 domain [53] (Table 3). This resulted in a loss-of-function effect leading to embryonic lethality at the time of blastocyst implantation for all homozygous mice and a considerable proportion of heterozygous mice [53]. Conversely, Krusche and colleagues developed viable heterozygous and homozygous mutant *Dsg2* mouse lines by systemic removal of exons 4–6 without disrupting the open reading frame [54] (Table 3). The obtained mutant gene encoded a shorter DSG2 protein lacking most of the adhesive extracellular domains EC1 and EC2 involved in the homophilic and heterophilic intercellular protein–protein interaction. Mutant DSG2 normally located at IDs, although its expression level was lower than in the WT counterpart. This, together with the reduced adhesiveness, may therefore weaken the connection between neighboring cardiomyocytes, as shown by the enlargement of the intercellular cleft and the frequent complete dissociation of cardiomyocytes [54]. Consequently, homozygous mutant mice developed arrhythmias and cardiac insufficiency that manifested together with ventricular dilation and increased interstitial and focal fibrosis [54].

Furthermore, infiltrations of CD45+ immune cells were detected in hearts of these mutant mice, in particular in correspondence of regions of acute lesion with active ongoing reparative processes [55]. Of note, increased expression of cardiac stress and heart failure markers, such as *Ccn2*, *Gdf15*, and *Myc*, was reported for homozygous mutant mice, while no differences were observed between WT and heterozygous mutants, in line with the absence of any other pathological change [54]. Similar histological and ultrastructural features, as well as functional deficiencies, were obtained by restricting exon 4–6 deletion uniquely to cardiomyocytes [56] (Table 3).

While excision of exons 4–6 of *Dsg2* resulted in the production of a shorter protein, elimination of exons 4 and 5 caused a frameshift mutation with a premature stop codon and production of a mRNA then degraded by non-sense mediated decay [57] (Table 3). Heterozygous knockout mice carrying this deletion (*Dsg2*^mut/WT^) did not show a strong ACM phenotype at baseline but developed myocyte injury and redistribution of ID proteins in response to endurance exercise [57]. Pharmacological treatment with SB216763, an inhibitor of GSK3β, a key protein involved in the inhibition of the Wnt/β-catenin signaling pathway, improved cardiac function, myocardial integrity, and survival in *Dsg2*^mut/WT^-exercised mice, implicating a central role for GSK3β signaling in ACM pathogenesis and disease progression in response to exercise [57]. On the other side, homozygous *Dsg2*^mut/mut^ mice developed ACM with complete loss of DSG2 by the age of 16 weeks, when abnormal distribution of junctional proteins, inflammation, and biventricular fibrosis became evident [57]. Treatment with SB216763 restored cardiac function and proper localization of CX43, both altered in *Dsg2*^mut/mut^ mice, indicating a potential new therapeutic strategy for ACM as previously hypothesized in a transgenic zebrafish model [57,58].

Further evidence supporting the implication of GSK3β signaling in ACM pathogenesis emerged from crossbreeding *Dsg2*^mut/WT^ and *Dsg2*^mut/mut^ mice with a pre-established strain of *Gsk3b* knock-in mice. This line expressed GSK3β with a serine-to-alanine substitution in position 9, resulting in its constitutive activation [59]. Significantly, in *Dsg2* mutant mice, the presence of one or two copies of active *Gsk3b* mutant allele hastened fibrotic deposition and exacerbated cardiac dysfunction [57] (Table 3). Of note, previous studies hypothesized that calpastatin depletion and calpain-1 activation in exercised *Dsg2*^mut/mut^ mice might lead to GSK3β constitutive activation. This was supported by findings demonstrating that calpain activation could truncate GSK3β, removing an N-terminal inhibitory domain [60]. Furthermore, either phosphorylation by active GSK3β or Calpain 1-mediated cleavage affected AIF nuclear translocation, triggering DNA fragmentation and cell death [61] (Table 3). Additional studies performed by the same group on *Dsg2*^mut/mut^ mice aimed to define the role of inflammation in the pathogenesis of ACM. Indeed, powerful inflammatory mediators, including interleukin 1-β, interleukin 12, interferon-γ, and tumor necrosis factor-α, as well as chemotactic molecules, were highly expressed in mutant hearts compared to WT [62]. Moreover, the nuclear factor-kB (NFkB) signaling pathway, known to be involved in cellular inflammatory responses, appeared to be active in the murine model as assessed by the increased expression and nuclear localization of phosphorylated RelA/p65 in *Dsg2*^mut/mut^ hearts compared to WT. Treatment with Bay-11-7082, a small molecule inhibitor of NF-kB pathway, over a period of 8 weeks, not only attenuated the production of inflammatory cytokines and reduced the number of infiltrating immune cells in *Dsg2*^mut/mut^ hearts but also prevented the development of ACM features [62].

Fu and colleagues took advantage of the same murine model to test a different pharmacological intervention aimed at reducing proinflammatory macrophages [63]. Starting from single-cell RNA sequencing of myocardial samples from ACM patients, the authors highlighted an enrichment of pro-inflammatory macrophages characterized by the expression of Nod-like receptor protein 3 (NLRP3), which was suggested to play a role in the inflammatory response and was proposed as a possible target. Consequently, *Dsg2*^mut/mut^ mice were treated over a 4 week period with either MCC950, a compound shown to inhibit NLRP3 activation, or PBS. Impaired ventricular function, increased cardiac fibrosis, and accumulation of pro-inflammatory macrophages became evident in the PBS-infused group, while these changes were alleviated in MCC950-treated mice [63]. Taken together, these results raised the possibility that targeting immune signaling could represent a novel approach for ACM therapy.

A different ACM knock-in mouse model was developed by Schinner and colleagues by introducing a single point mutation driving the substitution of the tryptophan in position 2 with an alanine [64] (Table 3). Homozygous mice (*Dsg2-W2A*^mut/mut^) developed functional and electrical abnormalities coupled with widened intercellular space at the IDs and fibrotic accumulation. Heterozygous *Dsg2-W2A*^mut/WT^ mice presented a milder phenotype, mostly restricted to the right ventricle. RNA sequencing performed on both *Dsg2-W2A*^mut/WT^ and *Dsg2-W2A*^mut/mut^ mice and compared to transcriptomic data from ACM patients identified integrin β6 (*Itgb6*) as a common dysregulated gene. In mutant hearts, ITGB6 accumulated at the IDs forming heterodimers with integrin α5 (ITGAV), known to activate the TGFβ pathway. Accordingly, increased levels of phosphorylated active SMAD2/3 were detected together with upregulation of downstream targets. The involvement of the TGFβ signaling pathway in the fibrotic phenotype of the animals was further demonstrated by the significant downregulation of profibrotic markers achieved after treatment with a TGFβ receptor I inhibitor [64].

Different transgenic mouse models with a cardiomyocyte-specific overexpression of DSG2, either wild-type or with p.N271S and p.Q558* variations, have been generated in different studies with the aim of investigating the pathophysiological mechanisms underlying ACM development [65,66] (Table 3). Cardiomyocyte-specific overexpression of wild-type DSG2 in mice did not lead to any structural or functional abnormalities [65,66]. Conversely, transgenic mice overexpressing DSG2 with the p.N271S variation, which corresponds to the murine homologue of the human p.N266S variant detected in a family with ACM (c.797A>G nucleotidic alteration [67]), recapitulated the clinical features of ACM. These included sudden death at young age, ventricular arrhythmias, cardiac dysfunction, biventricular dilatation, and the onset of fibrosis starting at 6 weeks of age [65]. Remarkably, despite this phenotype, these mice showed no discernible alterations in the level or distribution of desmosomal proteins, desmin, or CX43. Further investigation into mice with varying levels of transgene expression revealed a dose-dependent dominant-negative effect of the mutation [65]. In mice exhibiting low expression of the transgene, characterized by a delayed disease onset, ID widening coincided with the onset of conduction slowing, preceding cell injury, inflammation, and fibrotic replacement. Furthermore, evidence of an interaction between DSG2 and the cardiac channel Na_V_1.5 was established, suggesting that aminoacidic substitutions in DSG2 might induce a conformational change affecting the functional interaction between the two elements, subsequently reducing Na^+^ current [68]. Overall, these findings support the idea that conduction abnormalities could develop in patients carrying pathogenic variations in ACM genes even in the absence of structural changes, challenging the initial assumption that such abnormalities were merely secondary phenomena.

A distinct transgenic model, referred to as Tg-hQ13, was developed, featuring cardiomyocyte-specific overexpression of a truncated human DSG2 cDNA, mirroring the c.1672C>T nonsense mutation identified in ACM patients (p.Q558*) [69]. This model showed ultrastructural alterations of IDs [66]. In Tg-hQ13 mice, the overexpressed truncated form of human DSG2 failed to reach the IDs and instead localized within the cytoplasm. Interestingly, while loss of cardiomyocytes and signs of fibrosis became evident at 12 months of age, the Wnt/β-catenin signaling pathway appeared to be downregulated even prior to the emergence of histological alterations, as assessed by the reduction of β-catenin in its active form (ABC) and some downstream targets already at 3 and 6 months [66]. Moreover, 24 microRNAs were dysregulated in transgenic mice compared to controls. Target prediction for the most altered microRNAs, which were also highly conserved between different species, identified genes involved in the regulation of the Wnt/β-catenin pathway [66]. Moreover, by crossing Tg-hQ13 mice with an Hic1 tdTomato-reporter line, Rossi’s group identified heart-resident PDGFRα+ SCA-1+ cFAPs and elucidated their direct contribution to the development of cardiac fibro-fatty infiltration [70].

More recently, studies involving cardiomyocyte-specific *Dsg2* null mice (CS-*Dsg2*^−/−^), developed by Lin and colleagues, helped to get insight about alterations of lipid metabolism in ACM pathological scenario [71] (Table 3). CS-*Dsg2*^−/−^ mice showed ventricular dilation, impaired contractile function, and accumulation of lipids within cardiomyocytes. The model also presented downregulation of PPARα, a transcription factor promoting the expression of genes related to fatty acid uptake and oxidation, which acts as downstream effector of the mTOR-4EBP1 axis, essential for cardiac energy supply and normal heart function. Coherently, decreased phosphorylation of mTOR and 4EBP1 was observed in CS-*Dsg2*^−/−^ mice and was further exacerbated by the mTORC1 inhibitor rapamycin, leading to enhanced cardiac hypertrophy and lipid accumulation. Similarly, siRNA-mediated knock-down of *Dsg2* in HL-1 cells led to downregulation of mTOR-4EBP1 axis and β-oxidation pathway. Conversely, overexpression of both mTOR and 4EBP1 in HL-1 cells rescued fatty acid oxidation, while in vivo reactivation of PPARα by fenofibrate significantly alleviated the lipid accumulation and restored cardiac function [71].

The model exhibited activation of both canonical and non-canonical TGFβ signaling pathways, as evidenced by the substantial phosphorylation levels of both SMAD3 and STAT3 [72] (Table 3). The latter is known to sustain ECM homeostasis through regulation of collagen production and secretion by fibroblasts [73]. In vitro studies on HL-1 cells showed that *Dsg2* silencing induced the activation of STAT3 and subsequent expression of fibrotic markers; these effects were abolished by siRNA knock-down of *Stat3*. In mice, reactivation of PPARα reduced STAT3 phosphorylation and expression of fibrotic markers implying that the observed PPARα downregulation not only contributes to lipid accumulation but also to cardiac fibrosis [72].

**Table 3:** DSG2 murine models for ACM and corresponding features.

Gene	Experimental Model	Alterations	Refs.
*Dsg2*	*Dsg2* KO mice lacking exons 7–8	Embryonic lethality	[53]
	*Dsg2* KO mice lacking exons 4–6	Cardiac fibrosis Inflammatory infiltrates Ventricular dilation Arrhythmias and cardiac insufficiency	[54,55]
	Cardiomyocyte-specific *Dsg2* KO mice lacking exons 4–6	Aberrant CX43 localization Cardiomyocyte necrosis Cardiac fibrosis Functional alterations	[56]
	*Dsg2* KO mice lacking exons 4–5	Abnormal localization of junctional proteins Cardiac fibrosis Inflammation GSK3β constitutive activation ↑ NFkB signaling pathway	[57,61,62]
	*Dsg2* KI mice	Wide IDs Cardiac fibrosis Echocardiography and ECG abnormalities ↑ TGF-β signaling pathway	[64]
	Tg mice with cardiomyocyte-specific overexpression of DSG2 with p.N271S variation	Cardiac fibrosis Ventricular dilation Cardiac dysfunction Ventricular arrhythmias	[65,68]
	Tg mice with cardiomyocyte-specific overexpression of human DSG2 with p.Q558* variation	Decrease in size and number of desmosomes Cardiac fibrosis ↓ Wnt signaling	[66]
	Cardiomyocyte-specific *Dsg2* null mice	Lipid accumulation Cardiac fibrosis Ventricular dilation Impaired contractile function ↓ PPARα ↓ mTOR-4EBP1 axis ↑ TGF-β signaling pathway	[71,72]

## 6. Murine Models for ACM-Associated Desmocollin 2 Variants

Alterations in the desmocollin 2 gene (*DSC2)* have been identified in 1–15% cases of ACM patients [7].

DSC2 is a transmembrane cadherin with structure and function similar to DSG2 with which it interacts in desmosomes and *area composita* [9].

Among all the desmosomal genes involved in ACM, *DSC2* is the least investigated using animal models. A study performed by Hamada and colleagues demonstrated that the deletion of glycine in position 790 of DSC2, corresponding to the nucleotidic c.2368_2370del variation [74] found in some ACM patients, was insufficient to induce ACM in a knock-in murine model [75]. Indeed, neither heterozygous nor homozygous mice showed structural and functional defects or lethal arrhythmias, although homozygous mice revealed a slight left ventricular dysfunction with aberrant Ca^2+^ release [75] (Table 4). On the contrary, cardiomyocyte-specific overexpression of human WT *DSC2* in mice caused severe biventricular cardiomyopathy, with cardiomyocytes necrosis, patchy fibrotic replacement, and activation of acute inflammatory processes [76] (Table 4). Furthermore, delocalization and reduction of mRNA and protein levels of DSG2, PG, and DSP were observed in fibrotic areas but were preserved in unaffected regions of the myocardium [76]. These two models highlighted that, even if DSC2 and DSG2 play a comparable function in desmosome assembly, knockout or overexpression of the corresponding genes gives rise to completely opposite outcomes in terms of ACM features development. This could suggest that the two cadherins may have additional different functions which have not been investigated yet.

Notably, Mazurek and colleagues exploited the regulatory role of the microRNA-130a (hereafter miR-130a) on the levels of ID proteins [77]. Transgenic mice with cardiomyocyte-restricted overexpression of miR-130a were previously reported to develop left ventricular dysfunction and exhibit premature ventricular contractions [78]. Concurrently, both DSP and CX43 were drastically reduced and in vitro experiments in NIH 3T3 fibroblasts corroborated that miR-130a functioned as a translational repressor of both proteins [77,78]. Additionally, fibrosis, lipid accumulation, and increased myocyte cell death were observed at the cardiac level, overall suggesting that development of ACM features may arise even in absence of direct genetic alterations in ID genes [77].

**Table 4:** DSC2 murine models for ACM and corresponding features.

Gene	Experimental Model	Alterations	Refs.
*Dsc2*	*Dsc2* KI mice	No structural and functional defects Slight LV dilation in homozygous G790del mice Aberrant Ca^2+^ release in homozygous G790del mice	[75]
	Tg mice with cardiomyocyte-specific overexpression of WT DSC2	Myocardial necrosis Fibrotic replacement Severe cardiac dysfunction	[76]

## 7. Murine Models for ACM Associated with Plakoglobin Variants

Heterozygous variants in the *JUP* gene can be detected in 1% of ACM patients [7], while homozygous variants in the same gene were found in individuals affected with Naxos disease, a cardiocutaneous disease characterized by ACM, woolly hair, and palmoplantar keratoderma [79].

PG, also known as γ-catenin and encoded by *JUP*, is an important component of both desmosomes and *area composita* and is a member of the armadillo protein family [80]. PG interacts with DSP through its central arm domain containing 12 arm repeats, as well as with the desmosomal cadherins via its N-terminus [15].

Several studies support the crucial role of PG in cardiac structure and function.

Complete loss of PG in murine models led to embryonic lethality at E12.5 due to cardiac developmental defects and wall thinning [81] (Table 5). Ultrastructural studies on the same mutant model reported an important reduction in the number of cardiac desmosomes [81].

In contrast, heterozygous *Jup*-deficient mice were viable, despite showing cardiac abnormalities, such as right ventricular dilatation and ventricular arrhythmias at rest [82] (Table 5). Endurance exercise consisting in daily swimming training for 8 weeks in these animals led to premature right ventricular dilatation and tachycardia. At the molecular level, typical junctional proteins, such as β-catenin and PKP2, were normally localized and concentrated. However, CX43 concentration was lower in heterozygous knockout mice compared to WT animals, possibly associated with reduced conduction velocity evident in this ACM model [82,83] (Table 5). Taken together, these results demonstrate that a reduced expression of PG is sufficient to develop some ACM features, which can be exacerbated by training, as observed in human patients.

Deqiang Li and colleagues generated a cardiomyocyte-specific *Jup* knockout model that lacks exons 3–5 and mimicks the condition observed in patients affected by ACM. This model exhibited cardiac fibrosis and dysfunction, ventricular dilatation, and ventricular arrhythmias [84] (Table 5). Moreover, enhanced apoptosis of cardiomyocytes, along with absence of typical desmosomes at the junctional level, were observed [84]. These occurrences were accompanied by the overexpression of TGFB1 and p-SMAD, supporting the link between TGFβ signaling upregulation and fibrogenesis in ACM [84].

Cardiac dilatation and fibrosis were observed also in a different cardiomyocyte-specific *Jup* homozygous knockout mouse model, lacking exon 1, along with reduced levels of DSG2 at IDs [85] (Table 5). Analyses performed in the same study hypothesized that adrenergic signaling may enhance cardiomyocyte cohesion via the recruitment of DSG2. These results revealed that the effects of β-adrenergic signaling on the cohesion of cardiac cells was dependent on PG expression, making this process of high medical relevance for the study of possible treatments for ACM [85].

The group of Radice developed a conditional mouse model with cardiomyocyte-specific *Jup* deletion obtained by treating 6–8-week-old floxed littermates with tamoxifen [86] (Table 5). Comparably to what was observed on the models described above, these mice displayed, 5 months after treatment, enlarged ventricles; focal cardiomyocyte loss replaced by fibrotic tissue; and, close to these areas, the presence of apoptotic cells. Moreover, *Jup* reduction resulted in significative downregulation of DSG2, PKP2, DSP, and CX43, along with decrease in the number of desmosomes and GJ plaques, even if no conduction abnormalities were detected [86]. Interestingly, the absence of PG at the cardiac level led to the upregulation of its homologue β-catenin, which in turn resulted in the activation of the Wnt pathway and increased expression of target genes such as *Myc*, *Ccnd1*, and *Fos* [86].

To validate the hypothesis of the role of β-catenin in the stabilization of GJs, Swope and colleagues generated a cardiomyocyte-specific double-knockout (DKO) murine model lacking both PG and β-catenin [87] (Table 5). These mutant animals exhibited enlarged ventricles, conduction alterations, and arrhythmias, as well as excessive collagen deposition accompanied by fibroblasts interspersed in the myocardium and increased presence of apoptotic cells. At the structural level, dramatic disruption of IDs and sarcomeres were detected in the myocardium of DKO mice, confirming the important role played by PG and β-catenin in the organization of desmosomes and *area composita* [87]. Furthermore, the arrhythmic behavior in DKO models was related to altered GJs, in particular to the critical reduction of CX43, together with a decline in size and area occupied by GJ plaques [87].

ACM is characterized by fibro-fatty replacement of the normal myocardium; however, none of the models described above displayed the presence of adipose tissue in the heart. To obtain novel insights into the adipose source in ACM, Lombardi and colleagues generated three sets of lineage tracer mice and showed that adipose cells derived from second heart field cardiac progenitors [88] (Table 5). Among these models, mice overexpressing at the specific level of cardiomyocytes the Flag-tagged full-length PG showed adipocytes and fibrotic patches in the heart. At the molecular level, the transgenic PG increased its interaction with the transcription factor Tcf712, which in turn showed a reduced interaction with β-catenin. In addition, the expression of the Wnt/β-catenin signaling target gene *Myc* was significantly reduced in the murine model compared to WT mice [88]. To further validate the role of PG in the adipogenic process, the same authors generated another transgenic murine model overexpressing in the cardiomyocytes either the WT or a truncated form of PG (c.23654del2) corresponding to the human pathogenic variant p.W680fs* (c.2038_2039delTG) [89] detected in patients affected with Naxos syndrome [90] (Table 5). In both cases, PG overexpression resulted in increased mortality in mice and enhancement of both adipogenesis and fibrogenesis. In contrast, only the truncated protein was absent at the desmosomal plaques [90]. Interestingly, adipogenesis was enhanced in cardiac progenitor cells isolated from the heart of animals of both lines, and it was reverted by forcing the activation of the canonical Wnt signaling by treating the cells with 6-bromoindirubin-3′-oxime, an inhibitor of GSK3β, further corroborating the link between PG, this pathway, and ACM [90]. Accordingly, the inhibition of GSK3β was tested as a possible therapeutic target in an ACM zebrafish model [58], as well as in transgenic mice expressing the same cardiomyocyte-specific c.2038_2039delTG variant in the *JUP* gene described above [57,89,90] (Table 5). The murine model showed focal areas of inflammation, increased apoptotic cardiomyocytes, and downregulation of CX43 and PG signals at IDs. Treatment for 6 weeks with the GSK3β inhibitor SB216763 resulted in the reduction of fibrosis and inflammation, improvement of cardiac function, and normal localization of junctional proteins [57]. In contrast to WT mice, in which GSK3β was mainly present in the cytoplasm, in ACM murine models, this protein was predominantly located at myocyte junctions, and its pattern distribution was normalized after treatment with the drug. The improvement in phenotypic manifestations in these murine models was probably due to the effect of SB216763 on GSK3β [57]. Moreover, as previously described in cardiomyocyte-specific heterozygous *Dsp* knockout mice [39], also in this model, the impaired assembly of IDs led to NF2 phosphorylation and pYAP membrane localization retention with consequent Hippo pathway activation and Wnt signaling suppression [40] (Table 5).

**Table 5:** PG murine models for ACM and corresponding features.

Gene	Experimental Model	Alterations	Refs.
*Jup*	Homozygous *Jup* KO embryos	Less developed heart Thin cardiac walls Reduced number of cardiac desmosomes	[81]
	Heterozygous *Jup* KO mice	Right ventricle dilatation and dysfunction exacerbated with exercise Ventricular arrhythmias exacerbated with exercise Low concentration of CX43 protein	[82,83]
	Cardiomyocyte-specific *Jup* KO mice lacking exons 3–5	Enlarged hearts Right ventricle dilatation Hypertrophic cardiomyocytes Enhanced apoptosis of cardiomyocytes Cardiac fibrosis Absence of normal desmosomes ↑ TGF-β pathway	[84]
	Cardiomyocyte-specific *Jup* KO mice lacking exon 1	Cardiac dilatation Cardiac fibrosis Reduced levels of DSG2 protein at intercalated discs Abrogation of positive effects of β-adrenergic signaling	[85]
	Cardiomyocyte-specific *Jup* conditional KO mice	Enlarged ventricles Focal areas of cardiomyocyte loss Inflammatory infiltrates Cardiac fibrosis Reduced junctional proteins expression at the intercalated discs Reduced CX43-containing gap junction plaques Reduction in the number and length of desmosomes ↑ β-catenin ↑ *Myc* and *Fos* ↑ c-MYC ↓ GSK3β ↑ Wnt/β-catenin signaling	[86]
	Cardiomyocyte-specific *Jup* and *Ctnnb1* conditional KO mice	Enlarged ventricles Increased number of apoptotic cells Fibroblasts interspersed through myocardium Collagen deposition Reduced junctional proteins levels Decreased area occupied by gap junction plaques Loss of IDs structures	[87]
	Tg mice with cardiomyocyte-specific overexpression of Flag-tagged PG	Cardiac fibrosis and adiposis ↓ Wnt/β-catenin signaling	[88]
	Tg mice with cardiomyocyte-specific overexpression of truncated or wild-type PG	Cardiac fibrosis and adiposis Enhanced adipogenesis in cardiac progenitor cells Molecular remodeling of IDs Nuclear localization of plakoglobin ↓ Wnt/β-catenin signaling ↑ Hippo pathway Abnormal localization of junctional proteins Inflammatory infiltrates	[40,57,90]

## 8. Conclusions

ACM is one of the major causes of sudden cardiac death [1]. Nonetheless, a full comprehension of the pathogenic mechanisms leading to its development is still missing.

Over the years, disease models harboring alterations in genes coding for components of the IDs highlighted not only the importance of these proteins in maintaining tissue function and integrity but also their contribution to several signaling pathways.

Of most relevance, perturbation of the Wnt/β-catenin signaling appeared in different ACM models to be a recurrent mechanism possibly contributing to the characteristic disease hallmarks. Alterations of the pathway were proposed to result from several factors impacting on β-catenin degradation, nuclear translocation, or interaction with TCF/LEF transcription factors. For instance, aberrant desmosomes can cause both the release of PG, then competing with β-catenin, and the activation of NF2 resulting in YAP phosphorylation, which in turn might interact with β-catenin blocking its nuclear internalization. As a consequence, the activation of the Hippo pathway has been proposed as a possible suppressor of Wnt/β-catenin. Notably, Xinβ, a recently identified component of IDs, has been shown to regulate the Hippo pathway by interacting with NF2; loss of this factor resulted in post-natal cardiomyocyte proliferation defects and cardiac abnormalities [91]. Additionally, GSK3β activation was reported in some ACM models and, since it is involved in β-catenin degradation, was identified as a possible therapeutic target. Combined studies testing a GSK3β inhibitor in mouse and zebrafish models resulted in improved cardiac function supporting the hypothesis of GSK3β signaling involvement in ACM pathogenesis. Similarly, a reciprocal relationship between PPARγ and canonical Wnt signaling has been demonstrated in models of ACM, where a direct interaction between PPARγ and β-catenin can promote β-catenin degradation [92].

The fibrotic deposition seen in ACM seems to rely also on the TGFβ signaling pathway whose activation stimulates the expression of ECM proteins and inhibits matrix metalloproteinases. Indeed, in some models described above, an up-regulation of the pathway was reported while a down-regulation of pro-fibrotic markers was achieved by the treatment with TGFβ receptor I inhibitor.

Overall, these reports not only offered insights into the complex mechanisms underlining ACM but also identified potential therapeutic targets that may warrant further investigation in future preclinical studies.

Besides the important advantages that murine models provide to the comprehension of ACM, it is still important to consider that they also present several limitations. For instance, it is crucial to mention that adipogenesis is not commonly encountered in mice, despite reports of lipid droplets accumulation in few murine models. Moreover, transgenic mice present intrinsic limits linked to the overexpression of mutant genes that does not reflect the human genetic condition. On the other side, knock-in and knockout animals represent a better system, since the pathogenic alterations can be introduced in heterozygosis or homozygosis, recapitulating patients’ genotypes.

It is worth mentioning that several in vitro systems have been created to compensate for certain limitations of in vivo models. Initial two-dimensional (2D) cultures of primary cardiomyocytes or immortalized cell lines have been recently replaced by human-induced pluripotent stem cells (hiPSCs), which can be derived from patients who harbor a pathogenic variant and differentiated into cardiomyocytes (hiPSC-CMs). Of note, studies based on hiPSC-CMs from ACM patients recapitulated some of the hallmarks of the disease, including elevated lipogenesis and apoptosis, therefore being useful to test drugs and treatment methods on a large scale. Additionally, hiPSCs can be employed to generate endothelial cells (hiPSC-ECs) and fibroblasts (hiPSC-FBs), which may be combined together to develop three-dimensional (3D) systems recapitulating more closely the complexity and heterogeneity of the heart. A complete overview on the application of hiPSCs in the context of 2D and 3D in vitro models for modelling cardiovascular diseases has been previously reviewed [93]. However, current in vitro models are still immature and fail to replicate the intricate architecture observed in an in vivo model, underscoring the necessity of integrating findings from both types of disease models. It is worth noting that mice serve as a complex study platform exploitable for analyzing functionality, risk factors, or limitations of innovative therapeutic approaches such as gene therapy treatments. Accordingly, some of the models described in this review were used to test the efficacy of AAV-mediated therapeutic approaches; this type of therapy could be decisive in patients presenting genetic variants resulting in haploinsufficiency, such as ACM patients manifesting alterations in *PKP2* gene. For example, PKP2 restoration carried out via AAV administration of the WT form of either the murine or human gene in knock-in or knockout mouse models previously characterized revealed positive effects, arresting of the disease, or prevention pathogenic manifestations [34,94,95]. Furthermore, the gene therapy approach demonstrated a dose-dependent efficacy in preventing the disease development when administrated before evident significant structural changes [96]; AAV administration after a few weeks of conditional *Pkp2* cardiomyocyte deletion, when structural effects have occurred, prevented further decline of heart functionality and reduced mortality [96].

Overall, both in vitro and in vivo models present advantages and limitations that might be mutually compensated. Nonetheless, their combination might represent a precious tool in order to gain an understanding of the fundamental mechanisms that drive the pathological features of ACM, therefore providing new opportunities to develop new targeted therapeutics.
